# Double layer acceleration of ions with differently charged states in a laser induced plasma

**DOI:** 10.1007/s00339-023-06840-6

**Published:** 2023-07-30

**Authors:** Xiang Yao, Christof W. Schneider, Nadezhda M. Bulgakova, Alexander V. Bulgakov, Thomas Lippert

**Affiliations:** 1 Research With Neutrons and Muons Division, Paul Scherrer Institut, 5232 Villigen-PSI, Switzerland; 2 HiLASE Centre, Institute of Physics ASCR, Za Radnicí 828, 25241 Dolní Břežany, Czech Republic; 3 Laboratory of Inorganic Chemistry, Department of Chemistry and Applied Biosciences, ETH Zürich, 8093 Zurich, Switzerland

**Keywords:** Pulsed laser deposition, Thin films, Plasma spectroscopy, Kinetic energy distribution

## Abstract

The electric field driven acceleration of plasma ions is an intrinsic effect in laser-induced plasma plumes and is responsible for the generation of high-energy ions. At high laser fluences (≥ 2 J/cm^2^), multiply charged ions are formed and affect the plume expansion dynamics. In this paper, we used kinetic energy-resolved mass spectrometry to investigate the relative abundance and kinetic energy distributions of singly- and doubly-charged ions produced by KrF-laser ablation of nine different oxide targets. The doubly charged metal ions with a lower mass-to-charge (*m*/*z*) ratio show narrow energy distributions at high average kinetic energies coinciding with the cutoff energies for the singly-charged ion distributions. The observation suggests that the recombination of higher charged ions plays a prominent role in the formation of the high-energy tail for singly-charged ions. The results are discussed in terms of component volatility and a dynamic double layer, where ions with different *m*/*z* values experience different accelerations.

Ion acceleration in an expanding plasma by a self-consistent ambipolar electric field has been discussed for a long time [1–12]. It was first proposed by Plyutto [1] for the explanation of supersonic ion velocities during the expansion of a highly dense plasma into vacuum. In an expanding plasma, electrons having much larger velocities as compared to ions escape beyond the main plasma body that leads to a violation of charge neutrality at the plasma boundary and generating an ambipolar electric field. The region where the charge quasi-neutrality is broken is called a double layer (DL). This refers to the layered structure of the plasma with an external electron-rich layer (negative space charge) followed by an ion-rich layer (positive space charge). The corresponding ambipolar electric field does not allow electrons in the negative layer to escape completely from the plasma plume by dragging them back to the positive layer, so that the overall charge quasi-neutrality of the plume is preserved. When the positively charged ions enter the electric field, they are accelerated toward the electron layer. As a consequence, electrons at the negatively charged layer are slowed down, while ions are accelerated. The energy of these electrons is then transferred to the kinetic energy of the ions [9, 13].

The existence of the double layer and the key role of energetic electrons forming the DL has been demonstrated in experiments with a collisionless plasma expanding in high vacuum in the presence of an external magnetic field [10]. In a plasma generated by a nanosecond laser, the pulse duration is sufficient for the initial plasma plume to absorb the laser light in inverse bremsstrahlung and multi-photon absorption processes to further ionize and break bonds of molecular plasma species. The absorption of laser light in the initial plume serves, therefore, as an energy reservoir that produces energetic electrons and sustains a long-living double layer [9, 13–15]. In our early works [16, 17], a phenomenological model of the dynamic double layer was proposed based on the energy analysis of singly charged ions in multicomponent laser-induced plasma plumes. According to the model, the light and heavy ions in the plasma plume separate due to different acceleration rates. During the expansion, light ions with higher velocities than heavy ions experience a shorter acceleration time as they cross the double layer region with the higher electric field faster while heavy ions stay in this region for a longer time. As a consequence, heavy ions obtain more energy than light ions due to the longer acceleration by the double layer field. Light ions, however, can often show a ‘high energy peak’ in their energy distribution (IED) because of their accumulation in front of the ablation plume after fast passing the acceleration DL region. Ions with a higher charged state have been reported to be present in laser-induced plasmas which contribute to the plume expansion dynamics [18–24]. In particular, the recombination of highly charged ions during the expansion are likely to affect the final energy distributions of lower-charge ions [18]. Therefore, to further develop the understanding of the dynamics of the DL [16, 17], we perform an energy analysis of doubly charged ions in the phenomenological model of DL acceleration to illustrate the complicated dynamics of ion expansion in laser-induced plasmas.

The ion energies were analyzed by an energy-dispersive quadrupole mass spectrometer (Hiden EQP–MS) in a vacuum chamber at a pressure below 1 × 10^–5^ mbar with a target-probe distance of 4 cm. A detailed description of the plume characterization system was presented elsewhere [17]. A KrF excimer laser (Lambda Physik LPX 300, 20 ns, λ = 248 nm) with a fluence of 2 J/cm^2^ was used for all the ablation experiments. A series of oxide targets (LiMn_2_O_4_, La_0.33_Ca_0.67_MnO_3_, LuMnO_3_, La_0.6_Sr_0.4_MnO_3_, YBa_2_Cu_3_O_7_, TbMnO_3_, EuAlO_3_, ScMnO_3_, SrTiO_3_) were ablated and the energy distributions of singly (M^+^) and doubly (M^2+^) charged ions in the ablation plume analyzed.

Figure 1 shows the comparison of the IEDs of M^+^ and M^2+^ ions for the ablation of La_0.33_Ca_0.67_MnO_3_. As it has been reported in [16, 17], the IEDs of La^+^, Mn^+^, and Ca^+^ have a peak at low energies and an energetic tail attached to this low energy peak, while O^+^ has two peaks, of low and high energies. Unlike the metal M^2+^ ions, O^2+^ is not detected in the plume, most probably due to its large ionization energy (35.1 eV). Very distinct IEDs for Ca^2+^ and Mn^2+^ have been obtained. Contrary to IEDs of singly charged ions, the distributions for Ca^2+^ and Mn^2+^ are very narrow and located close to the high energy end of the respective M^+^ IEDs. This type of IED for M^2+^ with only a high energy peak is considered to be representative of M^2+^ IEDs for all the plumes studied for the described ablation conditions.

**Fig. 1:**
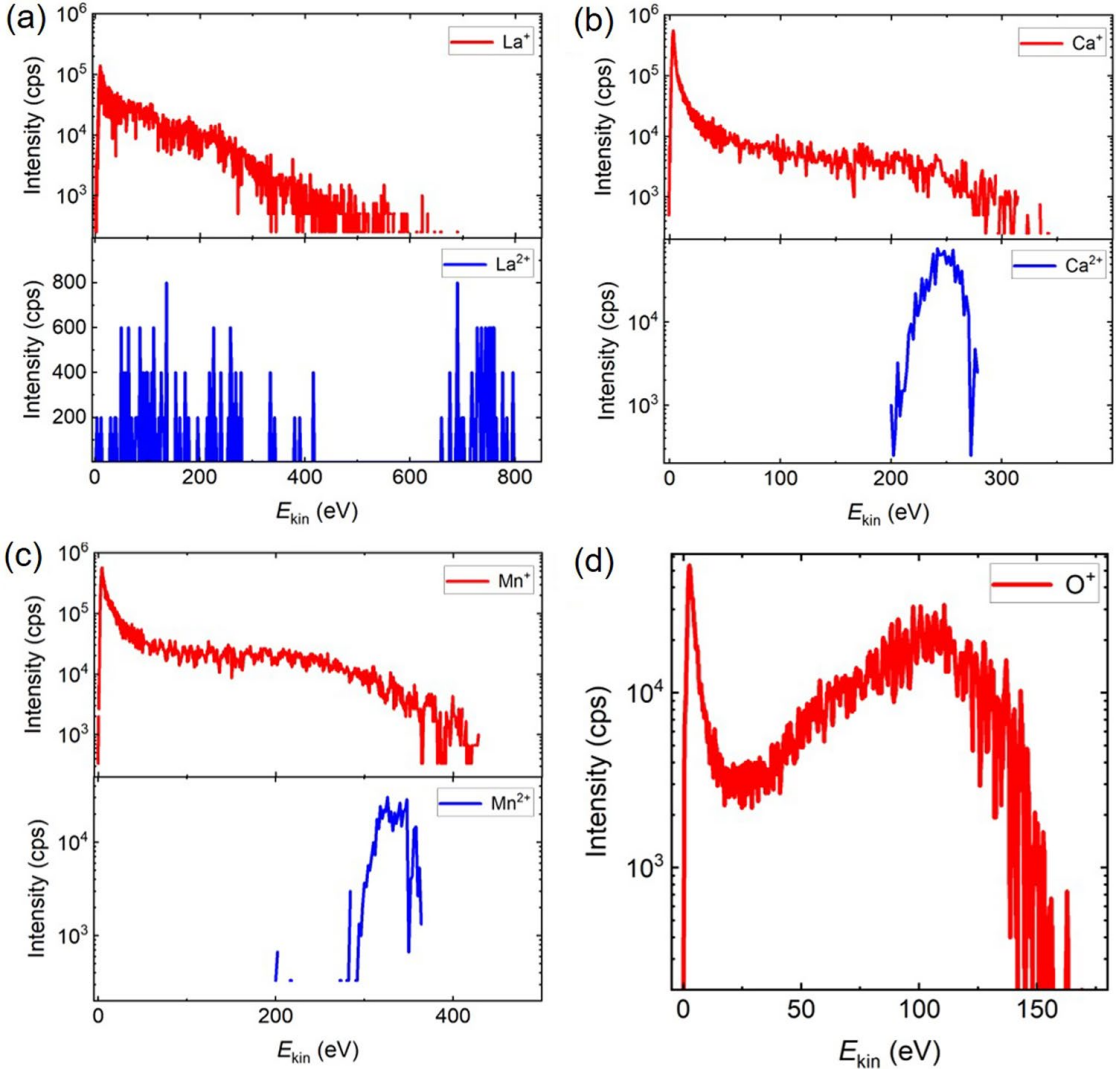
Comparison of the ion energy distributions of singly (upper row) and doubly (lower row) charged ions for the ablation of the La_0.33_Ca_0.67_MnO_3_ target. The O^2+^ ions are not detected in the plume. The intensity for the singly charged ions is plotted in the logarithmic scale to address the cutoff energies

For La^2+^, even though its ionization energy is much smaller than that of Mn^2+^ (11 eV for La^2+^; 15.6 eV for Mn^2+^), the measured amount when ablating La_0.33_Ca_0.67_MnO_3_ is very small, so that the La^2+^ signal shown in Fig. 1 is almost within the noise level of the measurement. When ablating LiMn_2_O_4_, no doubly charged ions have been found, even though the same ablation conditions have been used. To analyze the factors influencing the abundance of doubly charged ions in the plume, the relative amounts of M^2+^ ions (M^2+^/M^+^) in each plume produced by ablation of different targets are listed in Table 1 together with the corresponding ionization energies, and mass to charge ratios. The total ion counts were deduced from the overall integration of the corresponding energy distribution curves and the ratio of M^2+^/M^+^ was calculated thereafter. The threshold value for M^2+^/M^+^ is taken to be 1 × 10^–2^. Any smaller value is considered to be equal to 0. The M^2+^/M^+^ data of Table 1 are plotted in Fig. 2 as a function of the mass-to-charge ratio (for the cases when the M^2+^ signal of the lightest plume component is above the detection limit) to illustrate the trend of the relative ion abundances with increasing in ion mass.

**Table 1:** Measured relative amounts of doubly charged ions (M^2+^/M^+)^ in different plumes

Target	Ion	Mass to charge ratio (*m*/*z*)	Ionization energy for M^2+^ (eV)	Ionization energy for M^+^(eV)	M^2+^/M^+^
LiMn_2_O_4_	Li^2+^	3.5	75.6	5.4	0
	Mn^2+^	27.5	15.6	7.4	0
La_0.33_Ca_0.67_MnO_3_	Ca^2+^	20	11.9	6.1	3.36 × 10^–2^
	Mn^2+^	27.5	15.6	7.4	1.48 × 10^–2^
	La^2+^	69.5	11	5.5	0
LuMnO_3_	Mn^2+^	27.5	15.6	7.4	1.76 × 10^–1^
	Lu^2+^	87.5	13.9	5.4	0
La_0.6_Sr_0.4_MnO_3_	Mn^2+^	27.5	15.6	7.4	9.4 × 10^–2^
	Sr^2+^	44	11	5.7	1.2 × 10^–2^
	La^2+^	69.5	11	5.5	0
YBa_2_Cu_3_O_7_	Cu^2+^	32	20.3	7.7	0
	Y^2+^	44.5	12.2	6.2	0
	Ba^2+^	69	10	5.2	2.44 × 10^–2^
TbMnO_3_	Mn^2+^	27.5	15.6	7.4	8.05 × 10^–2^
	Tb^2+^	79.5	11.5	5.8	0
EuAlO_3_	Al^2+^	13.5	18.8	6	0
	Eu^2+^	76.5	11.2	5.7	0
ScMnO_3_	Sc^2+^	22.5	12.8	6.6	3.18 × 10^–2^
	Mn^2+^	27.5	15.6	7.4	5.78 × 10^–2^
SrTiO_3_	Ti^2+^	24	13.6	6.8	1.49 × 10^–2^
	Sr^2+^	44	11	5.7	1.02 × 10^–2^

All targets were ablated with a 248 nm laser at a fluence of 2 J/cm^2^

**Fig. 2:**
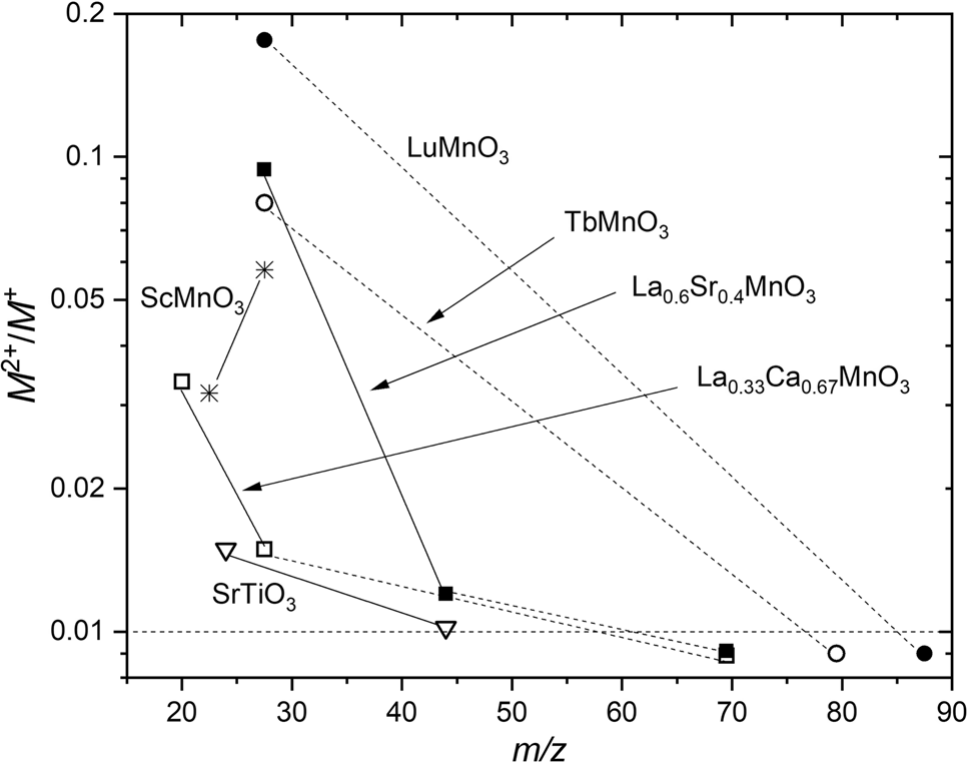
Ratio of M^2+^ to M^+^ ion signal intensities as a function of the mass-to-charge ratio of the doubly charged ions for different targets. The lines are to guide eyes. The M^2+^/M^+^ values which are below the detection limit of 0.01 are shown arbitrarily at M^2+^/M^+^ = 0.009 and are connected with measured points by dashed lines.to illustrate the general trends in ion abundances (see text for details)

According to Table 1, the relative abundance of M^2+^ ions in the plume does not show a one-to-one dependence on the ionization energy. However, some correlation of the M^2+^/M^+^ ratio with the mass-to-charge ratio (*m*/*z*) for the M^2+^ ions is noted. In general, the smaller *m*/*z*, the larger M^2+^/M^+^ becomes for most of the target materials investigated except for Li. When *m*/*z* is too high (e.g., Lu^2+^ in LuMnO_3_, Tb^2+^ in TbMnO_3_), M^2+^ is usually not detectable. The exceptions are ScMnO_3_ where *m*/*z* values for Sc and Mn are comparable and YBa_2_Cu_3_O_7_ where Cu^2+^ and Y^2+^ ions are not detected.

The correlation of the M^2+^/M^+^ ratio with the ionization energy in general is rather weak. If the ionization energy of the M^2+^ ion is very high [e.g., Cu^2+^ in YBa_2_Cu_3_O_7_ (20.3 eV) or Li^2+^ in LiMn_2_O_4_ (75.6 eV)], the ionization probability is too low and the M^2+^ signal cannot be detected. On the contrary, if the ionization energies of M^2+^ ions are relatively low, such ions can be easily produced (Ba^2+^, 10 eV) but are not detected in the plume (e.g., La^2+^, 11 eV). In addition, even for the same M^2+^ ablated from different target materials, the M^2+^/M^+^ ratio is different. For example, Mn^2+^ ions are abundant in most of the plumes but not detected for the ablation of LiMn_2_O_4_. Their relative amount M^2+^/M^+^ also varies in plumes with different compositions (Table 1).

A plausible explanation of the observed mismatch between the M^2+^/M^+^ ratio and the second ionization energy *I*_2_ can be seen in different volatilities of atomic species in a material matrix. It is known, that in the case of laser-irradiated compounds, more volatile species are ablated preferentially, thus leading to an enrichment of the irradiated surface by less volatile species [25–27]. Furthermore, time-of-flight measurements of vaporized species indicate that more volatile atoms/ions move in front of the plume ahead of less volatile species [27]. As a result, they are preferentially involved in the formation of the double layer and, thus, in the DL acceleration. The consequence of such vaporization dynamics is that less volatile species expand in the dense plume core. We can speculate that, even if they experience double ionization during the laser pulse action, they are swiftly recombining after pulse termination before the plume enters the collisionless expansion stage.

This concept looks to be fitting the observations of doubly charged ions summarized in Table 1. First, we mention that the oxide materials in the molten high-temperature state upon laser heating experience thermal dissociation enhanced also by photodissociation accounting for 5 eV laser photons whose energy exceeds or is comparable with the dissociation energies of oxide molecules. Atomic species being released due to dissociation leave the surface according to their volatilities. Among metals in the compound materials under study, lithium is the most volatile component [28], while manganese also demonstrates an enhanced volatility at increased temperatures [29]. Thus, the absence of doubly charged ions upon ablation of LiMn_2_O_4_ can be explained as follows. Lithium atoms and ions expand in the front of the plume. Due to an extremely high ionization potential *I*_2_, Li^2+^ ions cannot be observed at our relatively low laser fluence. As for Mn, its relatively low *I*_2_ points out the possibility of Mn^2+^ formation. However, being vaporized with delay with respect to Li and, hence, expanding in a relatively dense plume core, Mn^2+^ ions quickly recombine after the laser pulse termination.

Similarly, one can explain the absence of Y^2+^ and Cu^2+^ upon laser ablation of YBa_2_Cu_3_O_7_ as barium is well volatile at temperatures already above ~ 900 °C [30]. Having the lowest *I*_2_ value among YBa_2_Cu_3_O_7_ atomic species and moving in front of the ablation plume, it can exhibit doubly ionized ions, while Y^2+^ and Cu^2+^, if formed, experience recombination in the plume core. Among rare-earth metals, Lu, La, and Tb belong to a nonvolatile family of elements [31]. Thus, even being doubly ionized due to relatively small *I*_2_, they move behind the volatile Mn atoms and ions and recombine in a dense plume core before reaching the collisionless expansion stage. As for EuAlO_3_, both Eu and Al atoms are refractory [32]. However, it can be speculated that, due to the large difference in atomic masses, Al atoms and ions expand faster upon laser ablation and move in front of the ablation plume. Having a rather large *I*_2_ value, aluminum atoms do not experience a double ionization at our relatively low laser fluence, while Eu, if doubly ionized, recombines upon expansion in the plume core. However, the outlined effects of volatility for multicomponent materials ablation require an additional comprehensive investigation.

It should be mentioned that the time delays in laser vaporization of components with different volatilities may differ substantially for different compound materials. For example, upon nanosecond laser ablation of InP semiconductor, In atoms stop to be released from the irradiated surface quickly after the laser pulse termination, while phosphorus continues to be vaporized up to a microsecond timescale [33]. On the contrary, for the ablation of AuAg alloys, the time delay between the ablation of gold and silver is less than a nanosecond that, however, influences considerably the laser plume expansion dynamics [27]. The subtle effect of the volatility impact on the laser plasma expansion calls for further studies.

According to the double layer formalism [16, 17], ions are accelerated by the Coulomb force $\overrightarrow{F}=q\overrightarrow{E}$ ($\overrightarrow{E}$ is the local electric field). The acceleration is then $\overrightarrow{a}=\overrightarrow{F}/m=\frac{q}{m}\overrightarrow{E}=\frac{z\left|e\right|}{m}\overrightarrow{E}$ and thus is inversely proportional to the *m*/*z* ratio. Therefore, the *m*/*z* ratio is more appropriate for the description of the double layer acceleration than solely the ion mass. The separation of ions due to the double layer acceleration is then governed by the *m*/*z* ratio in the case of the presence of the multiply charged species. Thus, M^2+^ ions can cross the double layer considerably faster than the corresponding M^+^ ions, in a similar way when comparing light and heavy singly-charged ions.

As mentioned above, multiply charged ions can be formed in laser-induced plasma plumes under PLD conditions during the laser pulse action due to the heating of electrons in the inverse bremsstrahlung process followed by impact ionization and via the multiphoton ionization process [15, 34]. Ion recombination with electrons, e.g., from M^2+^ to M^+^, continuously proceeds in the plume core, where the collision frequency is substantial via three-particle and photo-recombination processes [15, 35]. However, if M^2+^ ions cross the double layer and enter the external collision-free region, where recombination is suppressed, they remain and are detected. For M^2+^ ions with a large *m*/*z* ratio (as well as with low volatility as discussed above), which cannot enter the collision-free zone, recombination converts them into M^+^ ions or finally to neutrals. This explains the observed correlation of the relative amount of the M^2+^ ions with the mass-to-charge ratio. It also explains why Mn^2+^ is not detected in the plume of LiMn_2_O_4_. The fast-moving Li^+^ ions cross the double layer quickly, thus reducing the DL field strength before a detectable amount of Mn^2+^ ions is able to enter the collision-free zone. Therefore, Mn^2+^ ions are not detected as they are mostly recombined in the plasma core.

One of the consequences of the recombination of multi-charged ions within the DL is that such ions serve as precursors for the energetic ions with a lower charge state [18, 36, 37]. The ions recombined from a high charge state after their efficient DL acceleration are detected at a lower charge state while keeping the kinetic energy gained upon acceleration. Hence, M^2+^ ions may contribute to the energy distribution of M^+^ ions, particularly in the energetic tail. This is the reason why the peak of M^2+^ ions always appear at the cutoff energy of the corresponding distribution of M^+^ ions (see Fig. 1). It should be also mentioned that the recombination rates of ions are proportional to *z*^2^ and *z*^3^ for photo- and three-particle recombination, respectively [34]. As a result, in the dense plume core, the doubly charged ions are rapidly converting into longer living singly charged ones, while they survive with higher probability in the front part of the plume, where the ablation plasma density and temperature drop dramatically within several hundreds of nanoseconds due to quasi-adiabatic expansion [38]. This explains the low-energy cutoff of the doubly charged ions.

In conclusion, we have found that the dynamic double layer accelerates ions according to their *m*/*z* ratios. Ions with a small *m*/*z* ratio gain large initial accelerations in the double layer and can rapidly cross the DL region with the high electric field. The consequence is the formation of high energy peaks in the ion energy distributions, which originate from the accumulation of accelerated ions with similar energies. M^2+^ ions also contribute to the energy distribution of M^+^ ions via recombination during plume expansion. Being accelerated in the high-field DL regions, where the collision frequency is still substantial, M^2+^ ions can recombine and then enter the external collision-free DL zone with reduced charge but enhanced energy. Different volatilities of the plume components can also affect the ion acceleration and recombination processes. Our study, therefore, widens the understanding of the origin and dynamics of high kinetic energy ions in laser-generated ablation plumes. This is of high importance as highly energetic ions can lead to both desired and undesired effects during the film deposition, such as a re-sputtering of deposited species, increasing film adhesion to the substrate, and subsurface implantation thereby creating defects in the growing films.

## Data Availability

The data that support the findings of this study are available within the article.
